# Ventricular Fibrillation Cardiac Arrest in Young Female from Diffuse Left Anterior Descending Coronary Vasospasm

**DOI:** 10.5811/cpcem.2019.9.43762

**Published:** 2019-10-14

**Authors:** Christopher J. Wilson, Eric Melnychuk, John Bernett

**Affiliations:** * Geisinger Commonwealth School of Medicine, Geisinger Medical Center, Department of Emergency Medicine, Danville, Pennsylvania; † Geisinger Commonwealth School of Medicine, Geisinger Wyoming Valley, Department of Emergency Medicine, Danville, Pennsylvania

## Abstract

This is a case of the most severe and potentially fatal complication of coronary artery vasospasm. We report a case of a 40-year-old female presenting to the emergency department (ED) via emergency medical services with chest pain. The patient experienced a ventricular fibrillation cardiac arrest while in the ED. Post-defibrillation electrocardiogram showed changes suggestive of an ST-elevation myocardial infarction (STEMI). Cardiac catheterization showed severe left anterior descending spasm with no evidence of disease. Coronary vasospasm is a consideration in the differential causes of ventricular fibrillation and STEMI seen in the ED.

## INTRODUCTION

Although previously thought to be rare, recent literature suggests that coronary artery vasospasm can lead to ventricular fibrillation.^1^ Recognizing this etiology is often difficult. Patients may not experience chest pain and may be younger and with fewer risk factors than those more usually diagnosed with acute coronary syndrome.^2^ Patients who experience ventricular fibrillation, often out of hospital, likely will present to the emergency department (ED) after out-of-hospital cardiac arrest or may experience cardiac arrest while in the ED. This is an important consideration to emergency physicians (EP) as this is a more prevalent cause of sudden cardiac death than previously thought.^1^,^3^,^4^,^5^ Given the risk of recurrence of arrythmia with spasm in patients who have previously experienced arrest, an EP can make a significant impact on the patient outcome by recognizing this as the causative etiology.^4^ Medical therapy with calcium-channel blockers and implanted cardiac defibrillators is the current method of preventing recurrence.^4^,^5^

## CASE REPORT

**Image 1:**
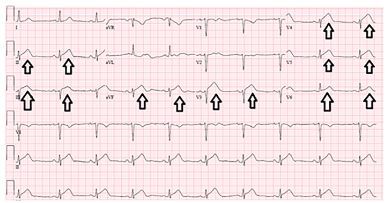
Emergency department electrocardiogram showing anterolateral and inferior ST elevation. Arrows indicate ST elevation.

A 40-year-old female presented to the ED with past medical history of non-ST elevation myocardial infarction (NSTEMI) from proposed coronary artery disease, hypertension, hypothyroidism, anxiety, depression, asthma, and tobacco use. The patient reported chest pain that started at rest and was associated with shortness of breath and radiation to the jaw and left arm. Although previously effective, nitroglycerin tablets did not relieve her pain, which prompted her to call emergency medical services (EMS). EMS administered 324 milligrams of aspirin and three nitroglycerin tablets to the patient. Upon EMS presentation to the ED, she became unresponsive and was noted to be pulseless. Cardiopulmonary resuscitation was initiated, with a heart rhythm of ventricular fibrillation. The patient was successfully defibrillated and had return of spontaneous circulation (ROSC). An electrocardiogram (ECG) (Image 1) immediately post ROSC showed ST elevations in anterolateral and inferior leads.

**Image 2:**
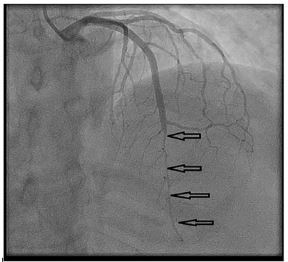
Mid and distal left anterior descending artery in severe vasospasm. Arrows indicate left anterior descending vasospasm.

The patient was taken emergently to the cardiac catheterization laboratory. The cardiac catheterization revealed the following: The left anterior descending artery (LAD) was without atherosclerotic disease; the LAD supplied the apex as well as inferior wall of the heart; and the proximal LAD was normal and large, while the mid and distal LAD were in diffuse spasm (Image 2).

The spasm was not present on the patient’s previous cardiac catheterization. The spasm did not respond to intracardiac nitroglycerin. The ejection fraction was reduced to 35%. Post catheterization the patient was started on a diltiazem infusion, and cardiac enzymes began trending down. Trans-thoracic echo two days later reflected a left ventricular ejection fraction of 45–49% with a moderate-sized apical wall motion abnormality with akinesis of the segments. She remained stable and was maintained on diltiazem in an attempt to prevent reoccurrence of the coronary vasospasm. She was fitted with a wearable defibrillator and discharged four days after arrival to the ED. Against cardiologist recommendation, the patient chose not to pursue an implantable cardiac defibrillator (ICD).

The patient had a follow-up with cardiology at one month, six months, one year and two years. She continued her beta blocker and calcium-channel blocker. Repeat echo at one-month post event showed a preserved left ventricular function with multiple wall motion abnormalities. She had no symptoms of chest pain since her event but did complain of shortness of breath with exertion. She again declined an ICD. The patient reported doing well overall and has optimized her medical management at subsequent follow-up visits.

## DISCUSSION

Coronary artery vasospasm leading to cardiac arrest was initially thought to be a rare event, but recent reports suggest it might be more common than previously reported.^1^,^4^,^6^ A recent study showed that coronary vasospasm accounted for 7% of out-of-hospital cardiac arrests.^1^,^4^ Another study has reported a rate as high as 11%.^6^

We identified three case reports in the literature. A 43-year-old had an out-of-hospital ventricular fibrillation arrest, and the initial cardiac catheterization showed nonobstructive disease. This patient developed chest pain one week later, and repeat catheterization showed severe spasm of the right coronary artery.^7^ Another case described the presentation of a 68-year-old without chest pain who had two episodes of syncope and then experienced a ventricular fibrillation cardiac arrest secondary to spasm of the right coronary artery.^8^ Finally, the literature describes a 46-year-old woman who had an out-of-hospital cardiac arrest secondary to vasospasm.^9^ Of note, a year prior she had chest pain; cardiac biomarker elevations and a catheterization showed normal coronary arteries. She was placed on a calcium-channel blocker, yet she experienced an adverse cardiac event one year later. Given the literature, our patient’s reported etiology of NSTEMI prior to the event presented here is called into question. It is possible that her prior NSTEMI actually was a presentation of coronary artery vasospasm without sudden cardiac death. Our case is unique in that the cardiac arrest occurred in in the ED.

**CPC-EM Capsule**

What do we already know about this clinical entity?

*Coronary artery vasospasm is a known cause of sudden cardiac death. Current literature indicates it may be a more common cause of cardiac arrest than was once thought*.

What makes this presentation of disease reportable?

*This patient was not the typical age or gender for sudden cardiac death*.

What is the major learning point?

*In patients outside of the typical age, gender, and risk-factor profile for sudden cardiac death, coronary artery vasospasm is a possible etiology*.

How might this improve emergency medicine practice?

*This case highlights an atypical patient at risk for sudden cardiac death, and the electrocardiogram and heart catheterization that demonstrated severe coronary artery vasospasm*.

When treating these patients, the benefit of an ICD should be evaluated. Research suggests that there are no reliable ways to predict risk of recurrence, and thus ICD placement with medications for vasospasm are advised.^5^ In our case, after the event it was highly recommended that the patient receive an ICD, which she declined. Additionally, she was given a wearable defibrillator but was not compliant with wearing it. Research has shown that patients who survive a coronary artery vasospasm that leads to a lethal ventricular arrhythmia have a very high risk of recurrence.^2^ Based on a novel risk-stratification system,^10^ the patient in our case is considered high risk and has an approximately 13% risk of recurrent adverse cardiac event. One study also suggests that the rate of recurrence continues to rise across the four years after initial coronary vasospasm.^3^

## CONCLUSION

This case adds to previously described literature by describing a ventricular fibrillation cardiac arrest secondary coronary artery vasospasm in the ED. Immediately following ROSC, our patient had ECG findings suggestive of STEMI, but with a cardiac catheterization that was suggestive of severe coronary artery vasospasm. Uniquely, this patient was followed for almost two years post-event and remained without neurological sequelae from the cardiac arrest event.
